# Factors Influencing Disability Inclusion in General Eye Health Services in Bandung, Indonesia: A Qualitative Study

**DOI:** 10.3390/ijerph16010023

**Published:** 2018-12-21

**Authors:** Manjula Marella, Fleur Smith, Lukman Hilfi, Deni K. Sunjaya

**Affiliations:** 1 Nossal Institute for Global Health, Melbourne School of Population and Global Health, The University of Melbourne, Melbourne, VIC 3040, Australia; smith.f@unimelb.edu.au; 2 Faculty of Medicine, Universitas Padjadjaran, Bandung, 40161 Indonesia; lukmanhilv@gmail.com (L.H.); dk_sunjaya@yahoo.co.id (D.K.S.)

**Keywords:** disability inclusion, eye health, inclusive eye health, universal health coverage, health services, access

## Abstract

The Inclusive System for Effective Eye-care (I-SEE) is a pilot project for disability inclusion in eye health in Bandung district of Indonesia. The aim of this research was to investigate factors influencing the introduction, i.e., adoption, implementation and continuation of I-SEE. A qualitative exploratory study was conducted by interviewing relevant stakeholders (n = 27) and users with disabilities (n = 12). A theoretical framework on the introduction of innovations in health care was used to guide data collection and thematic analysis. Factors related to the characteristics of the innovation (I-SEE) (e.g., infrastructure, equipment, engagement of people with disabilities, inclusive communication), service provider characteristics (e.g., motivation, attitudes, training), organizational characteristics (e.g., supervision, indicators, data), and the socio-political context of I-SEE (policy, motivation of users, family support, costs, transport) were essential for supporting the introduction process. Additionally, stakeholders proposed strategies for enhancing the introduction of I-SEE (e.g., awareness, collaborations). While there are specific disability related factors, most factors influencing the introduction of disability inclusive eye health were similar to introducing any innovation in general health care. Strategies for disability inclusion should be included from the planning phase of an eye health program and are reasonably simple to adapt.

## 1. Introduction

### 1.1. People with Disabilities and Their Eye Health Needs

The World Health Organization’s International Classification of Functioning, Disability and Health (ICF) defines disability as an outcome of an interaction between an individual’s health condition and the contextual (environmental and personal) factors the individual lives in [1]. According to this definition, disability is universal and can be experienced by everyone at some point in life either directly or indirectly. People with disabilities have similar health care needs as others but often face barriers in accessing health care, particularly in low and middle income countries [2]. These barriers include geographic location of services, inadequate transportation facilities, costs of getting to care, inadequate referrals, and negative attitudes of service providers and the community [3,4].

Disability inclusion is considered as one of the priority areas within the 2030 Agenda for Sustainable Development because it is now well recognised that the rights and needs of people with disabilities should be addressed for successful implementation of the Sustainable Development Goals. One of the objectives of the WHO Global Disability Action Plan 2014–2021 is to remove barriers and improve access to health care and programs for people with disabilities [5].

There are 1 billion people with disabilities globally [2]. There were 36.6 million people who were blind and 216.6 million people with moderate to severe vision impairment globally in 2015. It is estimated that these numbers are going to increase by 2020 with 38.5 million people blind and 237.1 million people having moderate to severe vision impairment [6]. The prevalence estimates of disability in Indonesia range from 1.4% to 21.3% based on different estimates [7]. In 2008, the Indonesian Central Statistics Agency estimated that physical and vision impairments were the most common types of disability [7].The Indonesian Population Census 2010 [8] estimated 9.73% disability prevalence based on modified Washington Group Short Set questions on Disability Statistics [9]. Using the question “Do you have difficulty seeing, even if wearing glasses?”, 3.44% of population reported seeing difficulties. There were 2.2% and 5.3% of people aged ≥21 years with blindness and moderate to severe vision impairment respectively in 2001 in Indonesia [10]. The prevalence of blindness in West Java based on the Rapid Assessment of Avoidable Blindness survey was 2.8% in 2014 [11].

**Figure 1:**
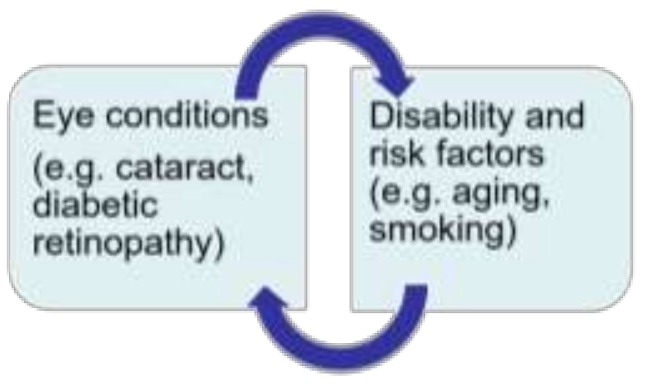
Cyclic relationship between eye conditions and disability.

Eye care is one critical area of health services where attention to disability inclusion is important. The relationship between eye conditions and disability can be described cyclic. For example, as shown in Figure 1, people with cataract can become vision impaired if they do not receive appropriate eye health care and could be disabled due to lack of participation in the community. On the other hand, people with disabilities, such as those with mobility impairments could develop cataract due to aging and other risk factors and experience further disability.

The 66th World Health Assembly endorsed the ‘Universal Eye Health: Global Action Plan 2014–2019’, which proposes universal and equitable access to services so that nobody is needlessly vision impaired ([12], p. 4). Supporting this Action Plan, a pilot project for disability inclusion in eye health, namely, the Inclusive System for Effective Eye-care (I-SEE) has been launched in Indonesia as part of the Australian Government’s aid program. In addition to this project, CBM and other international non-government organizations have implemented inclusive eye health programs in several low and middle income countries [13]. However, evidence on factors that influence the process of the adoption, implementation and continuation of disability inclusive eye health programs is limited.

### 1.2. I-SEE Project in Bandung

Disability inclusive health is “to ensure health systems recognize and accommodate the needs of people with disabilities in their policies, planning and service delivery” [14]. Indonesia has signed the United Nations Convention on the Rights for Persons with Disabilities (UNCRPD) in 2007 and ratified it by the Law No 19/2011. The Law No 8/2016 replaces the earlier Law ensuring the implementation of UNCRPD. The I-SEE project is designed in line with these laws promoting equal opportunities for people with disabilities in accessing eye health services.

Indonesian health system has both public and private health care providers. The public system is managed within a decentralized government system with central, provincial and district governments managing and resourcing different levels of health care. The Ministry of Health is responsible for the management and resourcing of some tertiary and specialist hospitals, such as the Cicendo National Eye Hospital (CNEH), which is a government tertiary level eye hospital. District and municipal governments are responsible for district, city and subdistrict hospitals and primary health care centres (*puskesmas*). *Puskesmas* are key for Universal Health Coverage or Jaminan Kesehatan Nasional (JKN) program [15].

The pilot I-SEE project in Bandung was initiated in 2014 to strengthen existing health systems to integrate eye health care and include people with disabilities. This four-year pilot project was undertaken in partnership with the District Health Office, as part of the government’s priorities to address non-communicable diseases and prevention of blindness. Cicendo National Eye Hospital is the major partner on this project, and Bandung district was chosen for piloting I-SEE within the district health care systems due to its proximity to the hospital for training and supervision.

Representatives of local Disabled Persons Organizations (DPOs) were engaged in the I-SEE project from its inception as an advisory team for planning, implementation and evaluation stages. The DPOs involved were Bandung Independent Living Center (BILiC), a cross disabilities local NGO and Persatuan Tunanetra Indonesia (Pertuni)—Indonesian Blind Union: Bandung chapter. In addition to these two DPOs, other local DPOs were involved in various stages of the project including to provide training and conduct audits of the eye health facilities. All DPO partners were provided training by CBM at the inception of the project on accessibility audit training and awareness raising activities.

In Bandung district, the I-SEE project was implemented in two government district hospitals (Majalaya Hospital and Soreang Hospital) and seven *puskesmas* (Cilengkrang, Cempakamulya, Ciluluk, Sudi, Kutawaringin, Rancabali, Ciparay). All together, these health centers covered eye health care at tertiary, secondary and primary levels. Eye nurses at *puskesmas* provide basic eye health screening, refraction and referrals to secondary or tertiary eye care at the district hospitals or CNEH respectively. Ophthalmologists at district hospitals prescribe eye glasses and perform cataract surgeries. All other complicated eye diseases including basic low vision rehabilitation services are provided at CNEH. I-SEE has formalized its partnership with BILiC and Pertuni to provide counselling and rehabilitation services for people with vision impairment later in 2017. Further, village health workers or cadres (who are part of the community health care system) and school teachers were also included in the I-SEE project and were trained by eye nurses at *puskesmas*. Village health cadres’ role was to support *puskesmas* to identify people with disabilities in the communities who might need eye health care and make referrals to *puskesmas*. Sometimes they even provided outreach activities such as ensuring people with disabilities are compliant with the treatment and ensuring their needs were communicated to *puskesmas* to facilitate appropriate referrals.

To promote disability inclusion in eye health, CBM Australia, an international non-government organization, has published a manual on ‘Inclusion made easy for eye health programs’ [16]. The manual has also been translated into Bahasa Indonesia. I-SEE was initiated by CBM in 2013 in Bandung district in West Java using the manual as a guidance tool.

The project involved addressing different determinants of inclusion such as: (1) training on disability concepts for staff at the CNEH in Bandung, staff of the District Hospitals and Puskesmas; (2) upgrading building accessible facilities (e.g., signage, ramps); and (3) referring people with disabilities attending the services to local DPOs for counselling and other services as needed. Selected staff from the CNEH and District hospitals along with eye nurses at *puskesmas* were trained on disability inclusion by DPO representatives and CBM staff. Selection of staff was made directly by the local clinics involved according to the roles and responsibilities of the staff involved. This initiative received further support with the introduction of the National Health Insurance Scheme (Jaminan Kesehatan Nasional, JKN) in January 2014, which covers the costs of health care including eye care for all people of poor socio-economic status [17]. As people with disabilities are at higher risk of being poor, JKN also facilitates access to health and eye care services through public hospitals for people with disabilities. People with disabilities referred from *puskesmas* to District hospitals can access subsidized or free eye glasses through the scheme.

### 1.3. Theoretical Framework

**Figure 2:**
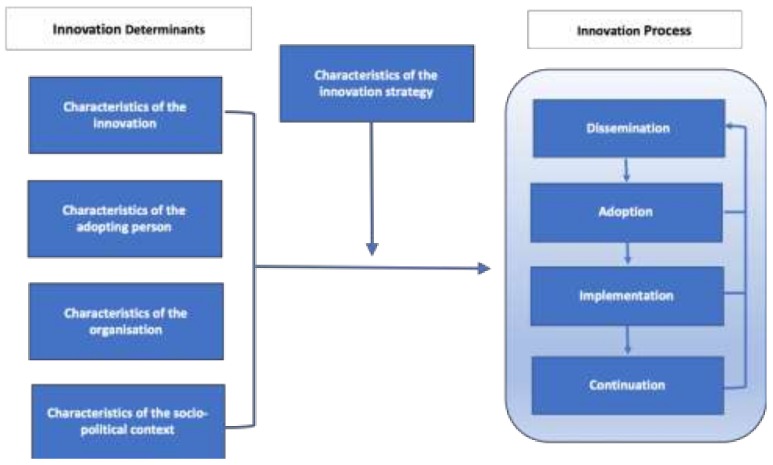
Framework representing the stages in innovation process and the determinants that influence the process. Derived from Fleuren et al. [18].

According to the theoretical framework (Figure 2) developed by Fleuren et al., the main stages in the introduction of any new interventions or programs (innovations) in health care are dissemination, adoption, implementation and continuation [18]. The framework also presents determinants/factors [19,20,21] that influence the transition from one stage to another, which are: (1) innovation characteristics such as the complexity or relative advantage of the innovation (here, I-SEE project); (2) the person adopting the innovation such as knowledge, skills and attitudes of the service providers; (3) organizational characteristics such as the organizational structure and processes for decision-making and monitoring; and (4) the socio-political context such as social and political environment the innovation is being introduced (e.g., patient characteristics, attitudes in the society, culture, community resources, policies and legislation).

These factors are also applicable for effective disability inclusive practices in health care generally and in eye care. A recent synthesis evaluation of CBM funded inclusive eye health project reports from eight low and middle income countries reported disability inclusive eye health requires: (a) involvement of multiple stakeholders, (b) existence of supportive policies and systems facilitating disability inclusion, and (c) adequate skills and knowledge of service providers [13]. Fleuren et al.’s framework provides an approach that considers disability inclusion in the context of the whole health care system, and thus provides a more comprehensive perspective on disability inclusion practices. It is critical to understand the factors influencing the process of introducing disability inclusion in health care as a first step to measure the effectiveness of disability inclusion.

Lack of evidence to inform disability inclusive practices is a major challenge in the development sector. The I-SEE pilot project in Indonesia provided an opportunity to understand the factors that influenced the processes of disability inclusion in I-SEE. This research was undertaken to explore how this new model of eye health care was adopted in Indonesian health system using Fleuren et al.’s framework. This study did not attempt to evaluate the outcomes of the I-SEE project or investigate how beneficial the project has been to the users with disabilities.

The aim of this research was to investigate the factors influencing the disability inclusive eye health practices (I-SEE project) in mainstream eye health care in Indonesia. Specific research questions were:

1) What are the factors influencing the introduction of disability inclusion in their eye health program as perceived by stakeholders?
2) Which factors are perceived by stakeholders as specifically important to the adoption, implementation and continuation of disability inclusion in their eye health program?
3) What are the perceived facilitators and barriers for users with disability to access eye health care through I-SEE?

## 2. Materials and Methods

A qualitative study was conducted to investigate factors influencing the introduction of the I-SEE project in Bandung district by interviewing relevant stakeholders and people with disabilities. Data collection was undertaken between April and June 2017. Ethics approvals were obtained from the Human Research Ethics Committee at the University of Melbourne and Health Research Ethics Committee at Universitas Padjadjaran. Permission from district health office was also sought as needed.

A total of 39 participants were included in the study (Table 1). Stakeholders working in different roles in I-SEE were purposively selected in consultations with the local advisory committee to allow identification of different factors. Stakeholders included eye care practitioners and senior officials at CNEH and District Hospital eye units, CBM Indonesia Office project officers, DPO representatives and village health cadres (equivalent to community health volunteer. Three groups of people with disabilities (physical, vision and hearing impairments) who have used eye care services from a selected District Hospital’s eye unit were purposively recruited through DPO networks. All participants provided written or verbal informed consent. For participants who were not literate or unable to read due to vision impairment, the consent form was read to them and their verbal agreement was recorded by the interviewer in front of a witness. This protocol was approved by the ethics committees (ID #1545948.).

Draft interview guides were developed for interviews with stakeholders and people with disabilities. The topics of the stakeholder interviews were based on the Fleuren et al.’s theoretical framework [18] comprising different stages and determinants of introducing disability inclusion in eye care. In case of interviewing people with disabilities, the topics were guided by the ‘Inclusion made easy in eye health programs’ manual [16]. The topics included the experience of using services, barriers and facilitators of accessing eye health services, and quality of care in terms of communication with service providers, information received on diagnosis and treatment, referral processes, and shared decision-making. All interview guides were finalised in consultation with the local advisory group, which included DPO members, CBM representatives, and eye health service providers. The interview guides were translated into the local language and back translated into English. Each interview was conducted in a private location convenient for participants and took approximately 60 min.

All interviews were digitally audio-recorded, transcribed and translated into English locally by a bilingual translator. Thematic analysis was conducted by the Melbourne-based researchers using a mixed inductive and deductive approach to generate codes and themes from the data using Nvivo software. MM and FS coded the data independently and compared. In case of discrepancies, both researchers discussed and resolved the differences mutually. The findings were shared with the participants of this research in a workshop conducted in December 2017 and were validated by them.

## 3. Results

Factors perceived by stakeholders and people with disabilities as influencing the disability inclusion in I-SEE project are shown in Table 2. Factors are mapped to different stages of introduction (adoption, implementation and continuation). Stakeholders identified factors relating to the I-SEE project itself as determinants of its successful adoption, implementation and continuation. They reported that engagement of people with disabilities, accessible health service infrastructure, accessible methods of communication, the availability of human resources and equipment were all important characteristics of a disability inclusive eye health program. People with disabilities highlighted factors related to inclusive communication, resources, attitudes of service providers, support from families, costs and transport as critical for the implementation and continuation of the project. As there was a significant overlap in the factors identified based on the responses from both groups, we combined responses of both type of respondents when reporting results.

### 3.1. Characteristics of the Innovation/Factors Related to I-SEE Project

#### 3.1.1. Engagement of People with Disabilities

Engagement of people with disabilities was identified as a key factor in all stages, but particularly the adoption stage of the project. Involvement of people with disabilities in planning, delivering training and the implementation of the I-SEE project was identified as important to the project’s success. The employment of people with disabilities as staff within the project was seen to be beneficial by some stakeholders in better meeting the needs of people with disabilities.

“*Firstly, it is very crucial, when we talk about the people with disabilities, the most important thing is their involvement. When the training is done by people with disabilities directly, then it is more effective than just having people without disabilities as trainers. Also, the involvement of people with disabilities, for example, in the assessments, and then the implementation… even in the planning, it is very helpful.*”

(Project officer 2, female)

“*We recommend them (people with disabilities) to be hospital officials….so that they comprehend more when a person with disability goes there.*”

(DPO representative 4, male)

#### 3.1.2. Physical Accessibility of Eye Health Facilities

Stakeholders identified having physically accessible eye health facilities as both a necessary feature and a key achievement or outcome for health services implementing the I-SEE project. There was recognition among many stakeholders of the need for a safe and accessible environment for people with disabilities, particularly for wheelchair users and those with vision impairments to provide inclusive eye health care. Stakeholders provided examples of ramped access into buildings, allocated parking spaces for people with disabilities close to entrances, handrails, low registration tables, and accessible toilet facilities as being necessary to making facilities accessible.

Stakeholders identified that physically accessible services were crucial for providing quality eye health services and for the acceptability and continuation of the services by the users. A lack of physically accessible facilities was identified as a significant barrier to disability inclusive health care, for example having eye clinics on upper floors of buildings without lift access. Further, a lack of understanding of the purpose of some accessible features of the buildings was felt by some stakeholders to lead to misuse as quoted below blocking access for people with disabilities.

“*Because of lack of understanding about the function of the infrastructure for people with disabilities, the misuse happened. For instance, the ramp was covered by motorbikes or the flower pots.*”

(DPO representative 1, female)

Stakeholders reported that despite the internal features in the hospital being upgraded to be physically accessible for people with disabilities, external features which are out of their control can create barriers for accessing services. Creating awareness about the need for physically accessible infrastructure in the community and local government support to upgrade infrastructure around the hospital such as roads and sidewalks were identified as important for a successful disability inclusive program.

“*Wheel chair users cannot access sidewalk and it should be fixed by the local municipality. We tried to fix it but it was difficult because it can be changed anytime (by the local government).*”

(Health service official 8, male)

#### 3.1.3. Inclusive Communication

Inclusive communication was identified as a critical factor for the implementation and continuation of the project, particularly by the users with disabilities. Participants with vision impairment reported the current communication methods are inclusive enough for them to understand the system when they are receiving services.

“*When they were going to check my health, they introduced themselves first. They introduced the names of the doctors, securities and others. So, when we meet, we know each other. Since I cannot see, they introduced their names first.*”

(User with a vision impairment 4, male)

However, challenges were noted for communicating with patients with hearing and speech impairments. Some users with communication difficulties mentioned that they needed to be always accompanied by family. A participant with hearing impairment suggested that the service providers could directly communicate with them by writing information.

“*They should give us information in writing, but they don’t use that one. I cannot hear so they should communicate with me in writing… I need my husband to accompany me as they (doctors) speak with him directly.*”

(User with hearing impairment 3, female)

Although some of the service providers were trained on sign language skills through I-SEE, not many patients with hearing and speech impairment attended eye health centers, and those who attended did not know professional sign language. Most people bring their family members and use their help for communicating.

“*Patients did not know the language. The sign language was not known by the patients and they were always accompanied by their family who generally answer what we asked. Well there was no training for people with disabilities itself. So, why did I learn about sign language, if the patients themselves didn’t understand what we talk about?*”

(Health service official 9, male)

#### 3.1.4. Resources

Having enough numbers of trained eye health workers was identified as a factor impacting on the adoption, implementation and continuation of the project. It was commonly reported by stakeholders that there are not enough human resources to provide the necessary coverage of eye health services let alone the capacity to undertake additional duties or take the time to ensure their practices were disability inclusive. The need for increasing the numbers of specialist eye doctors, eye nurses and cadres was identified as critical for the continuation and expansion of the project to other areas.

“*It is really not enough because we just trained 400 cadres who understood about eye care, meanwhile the demand is for 4000 cadres, it’s still way too far from the target.*”

(Project officer 1, male)

Some service providers discussed that they found it difficult to juggle the duties of the I-SEE project with their other duties and the geographic area they were required to cover. These difficulties were because of a lack of enough eye health staff and also due to responsibilities for the I-SEE project on top of their regular workload.

“*I am alone, with a lot of duties and responsible for other areas. To achieve the highest success expectation, it is very difficult since it conflicts with other duties.*”

(Eye nurse 2, male)

Loss of knowledge and training in inclusive eye health care due to frequent staff changes and a lack of incentives for cadres were other important human resource related factors identified as barriers to the success and continuation of the project.

The availability of tools and equipment necessary for the provision of eye health care is a factor that many stakeholders identified as important for successful implementation of the project. As with accessible infrastructure, some identified that the I-SEE project had helped to facilitate the availability of some of these tools (e.g., microscope, cataract set, refractometer) where previously they had been lacking.

“*It’s hard to get eye treatment here (in the puskesmas) because they are limited in tools and equipment, but in Al-Ihsan hospital (a regional public hospital), they have the tools.*”

(User with a vision impairment 3, female)

### 3.2. Characteristics of the Adopting Person/Factors Related to the Service Provider

Characteristics relating to service providers including their motivations for working in the I-SEE project, their attitudes and training, were perceived as determinants in the successful implementation of inclusive eye health care.

#### 3.2.1. Motivation and Attitudes of Service Providers

Service providers acknowledged that their involvement in the I-SEE project was part of their job responsibilities. Many noted that their increased understanding on the challenges that people with disabilities face in accessing health services following the trainings they received keeps them motivated to do their job.

It was noted by both stakeholders and people with disabilities that the attitude of service providers plays a critical role in the implementation and continuity of the project. Negative attitudes from service providers when people with disabilities attend a facility could influence their decision to not access services further. It was also noted that people with disabilities are more likely to openly ask for help if the staff at the health care facilities show their willingness to support them. The attitudes of service providers were also linked to the training they have received on disability inclusion (see Section 3.2.2).

“*…the approach to the patients, don’t be like, ‘this is disabled patient’…Yes, the stigma hinders them to get the health service.*”

(Village Health Cadre 4, female)

As indicated by a user with hearing impairment, people with disabilities would prefer going to health care facilities where they feel well treated by the staff.

“*The officers’ service there (at another puskesmas) was a little bit different, moreover, I am poor and have disability. They were not used to treating a person like me, but the service from doctors, nurses, and officers at this puskesmas (that is involved with I-SEE) was good.*”

(User with a hearing impairment 17, female)

#### 3.2.2. Training

Training for service providers on disability inclusion was reported as an essential aspect for the success of the I-SEE project throughout the stages of the innovation. Stakeholders highly valued training provided at the beginning of the project, which provided an understanding of the concepts of disability, rights of people with disabilities and technical skills on interacting and communicating with people with disabilities. Involving members of DPOs as trainers changed perceptions of stakeholders on the capacity of people with disabilities. Further, using simulations during the training was considered valuable for learning practical skills needed for implementing inclusive eye health practices.

“*People with disabilities were the trainers. It was amazing for me, I assumed that they can’t do it, but they actually did it, that motivated me. So, people with disabilities who have problems to speak could speak and gave an amazing explanation. It was incredible; hence I am interested to help the I-SEE inclusion Program for the disabled people in hospitals.*”

(Health service official 5, male)

However, receiving training as a one-off only during the adoption phase was felt insufficient for providing the necessary skills for service providers on disability inclusion. Continuous trainings to refresh and upskill were recommended. Interaction with people with disabilities in some clinics was reported to be rare and therefore staff who were trained on disability inclusion often lost their skills as they did not get to practice in their day-to-day work.

“*Yes, learning to communicate with the people with disabilities can’t be done in a short time, the time is short, and after the training, it is rare to meet the people with disabilities, so, we forget it.*”

(Eye nurse 2, male)

Some stakeholders identified that there are several staff types involved in different levels of eye care (hospital and *pukesmas*) and around the district, and that giving training to only some selected staff is not effective. Although attempts were made to share the knowledge with other staff, it was reported that it was not always feasible. Some reflected that the knowledge sharing with other staff may not be as comprehensive as the trainings provided by experts. Another challenge highlighted was the need to train new staff when the trained staff move or leave the clinics.

“*Only few staff joined the training, so we had difficulty sharing everything we learnt in training with others because we were not experts on this. Finally, we made a simple concept, and we shared information every month in front of all staff in our routine meetings. We told them how to communicate with people with disabilities, how to use sign language, describe the procedure to treat people with disabilities. So, we just simply shared.*”

(Health service official 9, male)

### 3.3. Socio-Political Context

Stakeholders identified the social and political context of where the I-SEE project is implemented as critical for successful adoption, implementation and continuation of the project. Factors identified under this theme overlapped with other themes. Patients’ health seeking behavior and support from their families, costs associated with health care and transport related issues were social factors that influenced the I-SEE project. Political characteristics were related to policy and support from the government.

#### 3.3.1. Patients’ Health Seeking Behavior and Family Support

Stakeholders identified that involving patients in decision-making about the services they needed is a critical aspect of service delivery. Patients’ understanding of the services and their desire or willingness to undertake eye health related treatment were perceived important contributing factors for successful implementation and continuation of the project.

“*If the patients are willing to do it (undertake services), then the other factors like the facilities and the doctors (come into the picture). It depends on the patients. If the patients refuse it, the tools and the facilities will be useless.*”

(Village Health Cadre 3, female)

Service providers particularly reported that patients often fail to accept services related to cataract surgery and other eye treatments at referral centers because they are scared, poor and require someone to accompany them. Some service providers even reflected that older people do not prioritize their vision problems and therefore refuse to seek services.

“*The first factor which prevents them to do it is their own fear…They (patients) are not fully aware of the importance of eye health. When we do the screening, and identify that they are meant to be operated, sometimes, they refuse the surgery because they are scared, it is the problem.*”

(Health service official 4, female)

Stakeholders highlighted that family support was an important facilitating factor in the implementation and continuation of I-SEE. People with disabilities receiving eye health care are mostly dependent on their families to bring them to eye health facilities and for ensuring compliance with treatment at home.

“*Family involvement at home after they get health care will make effective and optimum continuum of care. It is impossible that services will be finished from one visit, so the role of family is very important to help, to support their own member of the family with disability.*”

(DPO representative 1, female)

Discriminative attitudes of family and community towards people with disabilities and their health needs was identified as a significant challenge for accessing eye health care.

“*Sometimes the patients’ families ignore people with disabilities, so support from the family and society is really necessary for better inclusion of people with disabilities (in health care).*”

(Health service official 5, male)

#### 3.3.2. Costs and Transport

Direct health costs related to purchasing eye glasses or undergoing cataract surgery and indirect costs associated with transport, accommodation and food for the patient with a disability as well as their accompanying person often discouraged people with disabilities to get eye health services. Poor people with and without disabilities in general were perceived to consider health care as a low priority due to affordability issues. It was reported that health care card/insurance may not cover all costs associated with purchasing glasses or cataract surgery and that patients must pay some amount out-of-pocket.

Transport to and from eye health care services was identified by most respondents as a significant factor in the provision of disability inclusive eye health services. Often large distances to services for people with disabilities was seen by many as a deterrence for accepting or being able to access services. Having clinics closer to the community to reduce distance and transportation barriers was identified critical for inclusive eye health projects.

“*According to my opinion, for instance, me as a man with eye disease, it’s really hard for me. I want that the service to be closer so that small people like us can afford that. But if any service is near, like this puskesmas, that’s good. We will routinely come here because it’s near.*”

(User with vision impairment 6, male)

Some stakeholders stated that a lack of accessible transport necessitates people with disabilities having a family member or cadre to accompany them to the eye health clinics. A lack of availability of accessible transport and related costs were significant barriers to people with disabilities receiving eye health care. In some cases, stakeholders reported that cadres provided or paid for transport for their patients to enable them to access services.

“*The problem is that when the patients say they are really poor, they will think at least they need transportation cost, and then they think about giving food for the person who accompanies them there.*”

(Village Health Cadre 3, female)

“*Then at that time I was recommended to go to Cicendo, but you know, the transportation is difficult, and the cost is quite expensive. The health treatment is free, but the trip is not affordable.*”

(User with a hearing impairment 2, female)

In some cases, it was reported that village health cadres and eye health nurses have provided outreach services to the homes of people with disabilities to proactively identify those requiring eye health care and facilitate their inclusion in eye health services, in an attempt to overcome some of the barriers relating to distance and transport.

“*I waited for the patients to come here [to the clinic] but the more frequent thing is that we conduct home visit. I know about these six people with disabilities from home visit and from the cadres, I visited them.*”

(Eye nurse 1, male)

#### 3.3.3. Policy

Policy related factors including government support, laws and regulations, and access to national insurance scheme were identified as being important to the success of I-SEE at all stages of the project. Government support at all levels, including local government leaders (e.g., Regents/mayors), was identified by many stakeholders to be a key factor needed for the project. It was even indicated that having certain mandatory protocols from higher offices to lower offices are necessary for implementing disability inclusive practices.

Several stakeholders felt that at a funding and policy level there are many competing health priorities for government and that it could be difficult to prioritize inclusive eye health programs.

“*The challenge is that we work with the government as a partner, for example the district health office has so many agendas and activities, and the eyes health is not their priority. It is difficult to make them commit to the program.*”

(Project officer 2, female)

Some stakeholders identified existing laws and regulations that promote health services being inclusive, particularly regarding the physical accessibility of buildings. However, there was a sense from some stakeholders that although laws exist in Indonesia that promote the needs of people with disabilities, they are not always enforced.

The availability of national health insurance was considered a facilitating factor for the success of the I-SEE project. However, the complex and lengthy processes required to obtain insurance was seen as a barrier. Stakeholders reported that some people with disabilities preferred applying for a poor certificate (certificate of inadequacy or *Surat Keterangan Tidak Mampu—SKTM*) from their local village leaders as it is faster to do this and would provide with similar benefits for accessing healthcare as the national health insurance. For those without national health insurance, it was identified that I-SEE service providers (e.g., CBM, cadres) often helped to facilitate the process of obtaining a poor certificate.

“*I do not use the national health insurance or the government insurance because it takes a long time. I prefer asking the letter from the neighborhood leader. The covering letter to the hospital from the neighborhood leader, the SKTM letter [Certificate of inadequacy in English] is faster.*”

(User with a vision impairment 2, female)

However, many stakeholders also identified limitations to what is covered under the government health insurance, especially in relation to meeting the needs of people with disabilities, and that often there are still out of pocket costs.

“*Our social insurance scheme does not cover assistive devices like wheelchair. It just covers the spectacles and hearing devices, but not the wheelchair. I don’t know if the white cane is included or not. So how will the health insurance scheme raise the awareness that this is people’s right? It is still related to health sector and must be included to the social insurance scheme.*”

(DPO representative 1, female)

### 3.4. Factors Related to Health Facilities Supported by I-SEE

Systems for monitoring the progress on disability inclusion at health facilities participating in the I-SEE pilot project were considered critical for successful implementation and continuation of the project. Both the funding organization and the implementation partner should work together in regular monitoring and evaluation of the processes, with the availability of data on disability being an important factor needed to achieve this.

#### 3.4.1. Supervision, Monitoring and Evaluation

Stakeholders identified that having clear supervision, monitoring and evaluation systems is necessary to measure the achievement of the project’s targets and indicators, and that this was particularly important to the continuation of the project. It was also noted that budget allocations and human resources are required to achieve the necessary monitoring systems.

Stakeholders discussed that monitoring was also important for the success, transparency and maintaining quality of the inclusive eye health services. Some of the indicators suggested included the number of people with disabilities accessing services, the satisfaction of people with disabilities who receive services and the number of staff trained in disability inclusion.

“*Monitoring also becomes part of the most important factor of I-SEE success…. We start monitoring by looking at the database of how many hospitals are used by people with disabilities.*”

(Health service official 5, male)

#### 3.4.2. Disability Data

Different stakeholders identified the need for reliable data on disability both for planning services and for monitoring the I-SEE project. It was reported that the data on people with disabilities is varied across different departments and administrative levels, which could affect how disability is prioritized by each department within their programs.

“*The numbers (on people with disabilities) are different when we compare the data from social ministry and government insurance data. And then, the data in each level of government until in the village office level are different. The data should be the same, whether it is in the central government level, province level, sub-district level, or village level.*”

(DPO representative 4, male)

To enable monitoring of disability inclusion in the eye health services, stakeholders identified that data on disability needs to be recorded in medical records and health information systems and that currently this was not routinely available. Lack of information on disability numbers was reported to be a challenge for reporting on the achievements of the project and to monitor whether targets have been met.

“*In fact, in Puskesmas or Hospital, there is no integrated system to record whether the patient is disabled or not…We have not been able to figure out how many people with disabilities received the health service…Data is very important, that’s what determines what services must be provided, or what has been given so far. According to the new disability laws, now it is required.*”

(Project officer 1, male)

#### 3.4.3. Indicators of Success

Given this research was undertaken before the endline evaluations were conducted on the pilot I-SEE project in Bandung, stakeholders were asked what they would consider indicators of successful implementation of the project. Stakeholders identified several indicators for successful implementation of the I-SEE project. These can be grouped into two key clusters of indicators—those relating to success in terms of the inclusion of people with disabilities, and those relating to success in terms of eye health. Interestingly, while the DPO representatives primarily highlighted the disability inclusion related indicators all other stakeholders identified eye health specific indicators.

Eye health service utilization indicated by the number of people with disabilities attending *puskesmas* and eye clinics and patient satisfaction with services were considered to indicate the successful implementation of disability inclusion in eye health programs. The presence of accessible infrastructure and staff who are trained and skilled to meet the needs of people with disabilities attending eye health services were considered by stakeholders to be further indicators of success.

“*The medical staff also are ready to treat, they understand and are capable to handle people with disabilities.*”

(DPO representative 1, female)

In addition, success of the project was identified by many stakeholders to be indicated by a reduction in blindness and increased treatment of cataracts in the community, an increase in eye screenings, continuity of care for patients from referral through to surgery and sustainability of the project in the pilot centers.

“*If this pilot project runs as according to our expectation in 2017, and if the model is sustainable it can be used as an example for other district or other regions if they want to apply this system especially for eye care.*”

(Project officer 1, male)

### 3.5. Strategies for I-SEE Project Development

Stakeholders identified two critical factors for introduction of the I-SEE project: creating awareness in the community and collaborations/partnerships among key stakeholders.

#### 3.5.1. Awareness

Creating awareness about health care needs of people with disabilities and about the project was identified as a key strategy for adopting the project. Some of the strategies suggested were using media and inviting key political personnel to the events organized by the project. Some stakeholders also emphasized the need for involving the local community in the planning of the project and training events so that there is more acceptance in the community.

“*I think the promotion of the program should be intensively done with the media. When there is a health program for people with disabilities, it has to appear more frequently in the media.*”

(DPO representative 4, male)

It was noted that awareness and understanding of the issues for accessing health services by people with disabilities at the District Health Office level simplified the processes for adoption of the project. Raising awareness about health care needs of people with disabilities in the community was also identified as one of the key achievements of the project.

“*The main achievement is that the society finally could accept the existence of people with disabilities, not only about the equal treatment in hospitals…. Indeed, this lack of awareness is a problem, so it must be improved, so this program makes people aware through this program.*”

(DPO representative 1, female)

#### 3.5.2. Collaboration and Partnerships

Multi-sectoral collaboration was emphasized by several stakeholders for facilitating adoption, implementation and continuation of the project. Specifically, networks and collaborations between health care facilities, village health cadres, government, local authorities, schools, DPOs, and CBM Indonesia were reported to be essential for the success of the project and for its future sustainability.

“*The supporting factors have to, indeed, be from the cross-sector. We should cooperate with the sub-district, village offices, schools, and then from the community representatives. Without this cooperation it will be very difficult.*”

(Eye nurse 2, male)

Networks and collaborations between service providers were also noted as critical for timely and effective referral processes. For example, it can be frustrating for patients if the referral process is complex due to inconsistent information between village health cadres, health care facilities and government offices about how people with disabilities can access cataract surgery within the I-SEE project. Stakeholders recognized disability is a cross-sectoral issue and reported the need for cooperation between different government departments such as health, social, education and finance.

“*…. the people with disabilities are not only the health department responsibility. The government has to help them holistically with the departments which are related with the education, social, health, and financial departments, in case it is related with the budget.*”

(Health service official 5, male)

Further, the need for cooperation from private hospitals and the corporate sector was emphasized for future sustainability of the project. It was noted that government alone cannot support the health care needs of people with disabilities. Therefore, as part of the Corporate Social Responsibility, it was suggested that private hospitals and corporate companies could provide financial support needed for cataract surgeries.

To ensure the collaborations continue and are well supported it was suggested that formal agreements should be in place between the stakeholders involved and a monitoring system to support it. Regular meetings to ensure good communication between stakeholders was also indicated.

“*To improve the coordination and integration, there has to be commitment by all parties involved. Since there is agreement, we must be consistent and responsible to the determined commitment. Further, there must be a meeting and communication since the problem stems from poor communication, it is sometimes not optimum, those are the important factors.*”

(Project officer 2, female)

## 4. Discussion

The I-SEE pilot project addresses the WHO Global Disability Action Plan 2014–2019’s objective on removing barriers and improving access to health services and programs for people with disabilities [5]. As a pilot project in Bandung district, it provided the opportunity to learn what is feasible for a disability inclusive eye health system. This qualitative study explored stakeholders’ perception on the factors influencing the adoption, implementation and continuation of disability inclusive eye health practices in mainstream health care systems in Indonesia.

The factors identified in this research are in line with the factors identified for the introduction of any new systems/strategies in health care in general, and could be mapped into the components of Fleuren et al.’s framework [18,21]. Key factors identified for the introduction of I-SEE project can be represented at two levels: (1) generic to health systems and (2) specific to disability inclusion. Generic factors identified were costs, availability of transport for patients to and from health services, availability of a skilled/trained workforce/human resources, availability of necessary equipment and resources, and processes for monitoring and evaluation. These factors highlight the fact that disability inclusive systems are achievable with similar guidelines and principles as introducing any new system/strategy in health care and that parallel systems are not necessary.

The interaction between poverty, disability and health was highlighted by factors identified in this study. Findings indicate that poverty was a key deterrent for patients both with and without disabilities from seeking or accepting eye health services due to the unaffordability of direct costs (e.g., purchasing eye glasses, cost of surgery), and indirect costs (e.g., transport, food and accommodation for the individual and any accompanying person). These findings are similar to Baart and Taaka’s literature review on barriers to healthcare services for people with disabilities in developing countries [22]. Inability to access health care due to poverty increases the risk of poor health. Disability further exacerbates this interaction between poverty and health. People with disabilities are at greater risk of poverty due to lower employment rates and lower educational attainment [1] and this risk further leads to greater inequities in accessing health care and having their health care needs met, including those relating to eye health care, compared to the broader population.

While at higher risk of poverty, people with disabilities may also incur higher indirect health care costs. The need for an accompanying person to enable people with disabilities to attend health services was highlighted during the study and raised by DPO representatives during further stakeholder discussions. In addition to the transport and other costs for the accompanying person, their lost opportunities for income-earning activities creates a greater risk of household poverty, perpetuating the interaction between poor health and poverty for the person with a disability and their household.

Conversely, this study found that clinics that are close to communities and the availability of national insurance or a poor certificate, may reduce the financial burden of seeking health care, particularly for people with disabilities. Addressing the generic factors such as cost, transport, and health worker availability are therefore important for providing health care for “all” people, inclusive of people with disabilities.

Disability specific factors identified were related to inclusive practices—engagement of people with disabilities, accessible infrastructure, inclusive communication and attitudes of staff and community. These factors are relatively simple to include and address when introducing a new health service. These factors are best addressed by considering them in the planning and adoption of a health innovation such as inclusive eye health programs to ensure successful implementation and increase the likelihood of their continuation. For example, evidence suggests that the costs of providing accessible infrastructure from the outset adds little to building costs and is significantly cheaper than retrofitting [23,24]. Engaging people with disabilities, addressing negative attitudes and ensuring inclusive communication are all low-cost strategies and simply require implementers to adopt a proactive approach to disability inclusion from the outset of a health innovation.

The Universal Eye Health: Global Action Plan 2014–2019 aligns with the principles of universal health coverage [12]. Universal eye health coverage is impossible without ensuring marginalized groups such as people with disabilities are included in eye health programs. CBM’s guide, “Inclusion made easy in eye health programs” proposes practical strategies to strengthen disability inclusion in eye health programs [16]. Many of the factors identified in this study as being influential to the introduction of I-SEE align with those strategies—disability awareness, engagement and participation of people with disabilities including appointing to roles within eye health care programs, policies for disability inclusion, physical accessibility, accessible communication and costs of seeking healthcare.

This study identified that the majority of the factors perceived to be influencing the introduction of I-SEE pilot project were related to implementation and continuations stages. However, disability specific factors were particularly found to be influential at the adoption stage. This finding implies that specific strategies for promoting disability inclusion should be considered in the adoption stages of program introduction to achieve adequate coverage of services for people with disabilities.

The inclusive eye health programs so far have been implemented by international non-government organizations such as CBM and Sightsavers International. These organizations have closely worked with the local government systems and built capacity of eye health workers and supported building physically accessible facilities as seen in the I-SEE pilot model in Bandung. Morchen et al. [13] identified that training of local medical staff and government officials has resulted in better awareness about disability inclusion and rights of people with disabilities in various low and middle income countries. In most of these countries, as is the case of Bandung, the local governments are not resourced to undertake disability inclusive eye health programs. As indicated in the stakeholder meetings following this research in Bandung, the public health facilities involved in the I-SEE pilot project are willing to continue with disability inclusion activities with the increased capacity and awareness. Further, findings from endline project evaluations by CBM and based on the documented successes and lessons learnt from the pilot model, the inclusive eye health project is being expanded to other regions of Indonesia.

Two of the proposed strategies in the guide [16] are developing referral and support networks and access to disability specific/rehabilitation services including low vision services. The WHO Global Disability Action Plan 2014–2019 also proposes to strengthen the disability specific services related to rehabilitation, habilitation, assistive products and community-based rehabilitation [16]. One of the limitations of this study was not capturing information on referrals to disability specific services such as low vision services for those who are identified to have vision impairment. Some reference was made to the availability of assistive products for mobility, but not to services and/or assistive products for people with vision impairment. Further discussions with local stakeholders identified reasons for participants not reporting on disability specific services. Firstly, low vision services are only available in major cities in Indonesia, and they are not comprehensive. Availability of services only in Bandung means that not all people with vision impairment can access these services in the district. Secondly, the low vision devices are not covered under the government insurance scheme and therefore, they are not affordable to all people with vision impairment who need it. Finally, eye health practitioners are not aware of low vision services that are available and therefore, the referral processes are slow or sometimes do not happen. These challenges are not different to the current situation of low vision services in the Asia-Pacific region [25,26].

A further limitation of this study was that we could not recruit enough number of users with hearing and mobility impairments for this research primarily due to difficulties in tracking the users with different disabilities within I-SEE project data collection systems. Collection of disability-disaggregated data from the public health facilities included in the pilot I-SEE project has been challenging due to limitations with the information management systems. This challenge on collecting data on users with disabilities is not uncommon in many low resource settings where inclusive eye health programs have been implemented so far [13]. As indicated people with disabilities were purposively sampled through local service providers and DPOS due to time and resource limitations with the research study. Also, users with psychosocial disabilities and intellectual disabilities were not included as participants. Specific barriers and enablers to inclusion in eye health services for people with these impairments, and whether they differ from those identified in this study requires further exploration.

This research only focussed on the processes involved in the implementation of the project and was not aimed to evaluate the effectiveness of the I-SEE project. CBM Indonesia has conducted an independent evaluation of the pilot project separately. Therefore, this research did not collect information on the outcomes of the project and how beneficial it has been for the users with disabilities.

## 5. Conclusions

This study identified that perceived factors influencing the adoption, implementation and continuation of the disability inclusive eye health pilot project, I-SEE, were similar to the factors that would influence the introduction of any new intervention or program in a general healthcare system. While findings from this research are not surprising, they highlight that strategies for disability inclusion should be included from the planning phase of an eye health program and they are relatively simple and feasible to include. Information from this research is being used to develop strategies for replicating the I-SEE model in other location in Indonesia. Further, findings can support scaling up the disability inclusive health model to general health systems.

## Figures and Tables

**Table 1:** Participant types.

Participant Type	Total Number	Male	Female
	Stakeholders *		
Project officer	2	1	1
Village Health Cadre	5	0	5
DPO representative	4	3	1
Eye nurse	4	3	1
Senior officers at hospitals	9	4	5
Teacher	2	0	2
Other	1	1	0
Total stakeholders	27	12	15
	People with disabilities		
Physical impairment	4	3	1
Hearing impairment	2	1	1
Vision impairment	6	4	2
Total people with disabilities	12	8	4
**TOTAL**	**39**	**20**	**19**

* Four stakeholders had disabilities—2 × physical impairment, 1 × hearing impairment, 1 × vision impairment.

**Table 2:** Factors influencing the introduction of I-SEE project and the different stages.

Themes and Factors	Stages		
	Adoption	Implementation	Continuation
Characteristics of innovation/Factors related to I-SEE project			
Engagement of people with disabilities	x	x	x
Physical accessibility of eye health facilities	x	x	x
Inclusive communication		x	x
Resources	x	x	x
Characteristics of the adopting person/Factors related to the service provider			
Motivation of service providers	x	x	x
Attitudes		x	x
Training	x	x	x
Socio-political context			
Patients’ health seeking behavior and family support		x	x
Costs and Transport		x	x
Policy	x	x	x
Factors related to organization/I-SEE centers			
Supervision (M&E)		x	x
Disability data	x	x	
Indicators of success		x	
Strategies for introduction of I-SEE			
Awareness	x	x	x
Collaboration and partnerships	x	x	x

## References

[1] World Health Organization (2001). International Classification of Functioning, Disability and Health: Icf.

[2] World Health Organization, World Bank (2011). World Report on Disability.

[3] Drainoni M.-L., Lee-Hood E., Tobias C., Bachman S.S., Andrew J., Maisels L. (2006). Cross-disability experiences of barriers to health-care access consumer perspectives. J. Disabil. Policy Stud..

[4] Lawthers A.G., Pransky G.S., Peterson L.E., Himmelstein J. (2003). Rethinking quality in the context of persons with disability. Int. J. Qual. Health Care.

[5] World Health Organization (2015). Who Global Disability Action Plan 2014–2021. Better Health for all People with Disability.

[6] Bourne R.R., Flaxman S.R., Braithwaite T., Cicinelli M.V., Das A., Jonas J.B., Keeffe J., Kempen J.H., Leasher J., Limburg H. (2017). Magnitude, temporal trends, and projections of the global prevalence of blindness and distance and near vision impairment: A systematic review and meta-analysis. Lancet Glob. Health.

[7] Kusumastuti P., Pradanasari R., Ratnawati A. (2014). The problems of people with disability in indonesia and what is being learned from the world report on disability. Am. J. Phys. Med. Rehabil..

[8] United Nations (2015). Transforming Our World: The 2030 Agenda for Sustainable Development.

[9] Washington Group on Disability Statistics (2010). The Measurement of Disability Recommendations for the 2010 Round of Censuses.

[10] Saw S., Husain R., Gazzard G., Koh D., Widjaja D., Tan D. (2003). Causes of low vision and blindness in rural Indonesia. Br. J. Ophthalmol..

[11] Ratnaningsih N., Rini M., Halim A. (2016). Barriers for cataract surgical services in west java province of indonesia. Ophthalmol. Indones..

[12] World Health Organization (2013). Universal Eye Health: A global Action Plan 2014–2019.

[13] Morchen M., Bush A., Kiel P., Lewis D., Qureshi B. (2018). Leaving no one behind: Strengthening access to eye health programs for people with disabilities in 6 low- and middle-income countries. Asia Pac. J. Ophthalmol. (Phila).

[14] World Health Organization (WHO), United Nations Educational Scientific and Cultural Organization (UNESCO), International Labour Organization (ILO), International Disability Development Consortium (2010). Community-based Rehabilitation: Cbr Guidelines.

[15] Asia Pacific Observatory on Health Systems and Policies (2017). The Republic of Indonesia Health System Review 2017.

[16] CBM Australia Inclusion Made Easy in Eye Health Programs; 2013. cbm.org/disability-inclusive-eye-health.

[17] Ministry of Health Republic of Indonesia (2015). Indonesia Health Profile 2014.

[18] Fleuren M., Wiefferink K., Paulussen T. (2004). Determinants of innovation within health care organizations. Int. J. Qual. Health Care.

[19] Berwick D.M. (2003). Disseminating innovations in health care. JAMA.

[20] Greenhalgh T., Robert G., Macfarlane F., Bate P., Kyriakidou O. (2004). Diffusion of innovations in service organizations: Systematic review and recommendations. Milbank Q..

[21] Huijg J.M., van der Zouwe N., Crone M.R., Verheijden M.W., Middelkoop B.J., Gebhardt W.A. (2014). Factors influencing the introduction of physical activity interventions in primary health care: A qualitative study. Int. J. Behav. Med..

[22] Baart J., Taaka F. (2017). Barriers to healthcare services for people with disabilities in developing countries: A literature review. Disabil. CBR Incl. Dev..

[23] Australian Aid Program (2013). Accessibility Design Guide: Universal Design Principles for Australia’s Aid Program.

[24] Ostroff E. (2011). Universal design: An evolving paradigm. Univ. Des. Handb..

[25] Chiang P.P., Marella M., Ormsby G., Keeffe J. (2012). Critical issues in implementing low vision care in the asia-pacific region. Indian J. Ophthalmol..

[26] Marella M., Yu M., Paudel P., Michael A., Ryan K., Yasmin S., Minto H. (2017). The situation of low vision services in papua new guinea: An exploratory study. Clin. Exp. Optom..

